# Supplementary material for: Adenosine 2A receptor and TIM3 suppress cytolytic killing of tumor cells via cytoskeletal polarization
Source: Commun Biol. 2022 Jan 10;5:9. doi: 10.1038/s42003-021-02972-8 (PMC8748690; doi:10.1038/s42003-021-02972-8)
Supplement: Supplementary file 2 — Supplementary Information [file 42003_2021_2972_MOESM2_ESM.pdf]

Supplementary information for

Adenosine 2a receptor and TIM3 suppress cytolytic killing of tumor cells via cytoskeletal polarization

Grace L. Edmunds, Carissa C.W. Wong, Rachel Ambler, Emily J. Milodowski, Hanin Alamir, Stephen J. Cross, Gabriella Galea, Christoph Wülfing, David J. Morgan

Supplementary figures 1 and 2
Supplementary movies 1 through 4
Figure source files for figures 2 through 7

Supplementary Figures

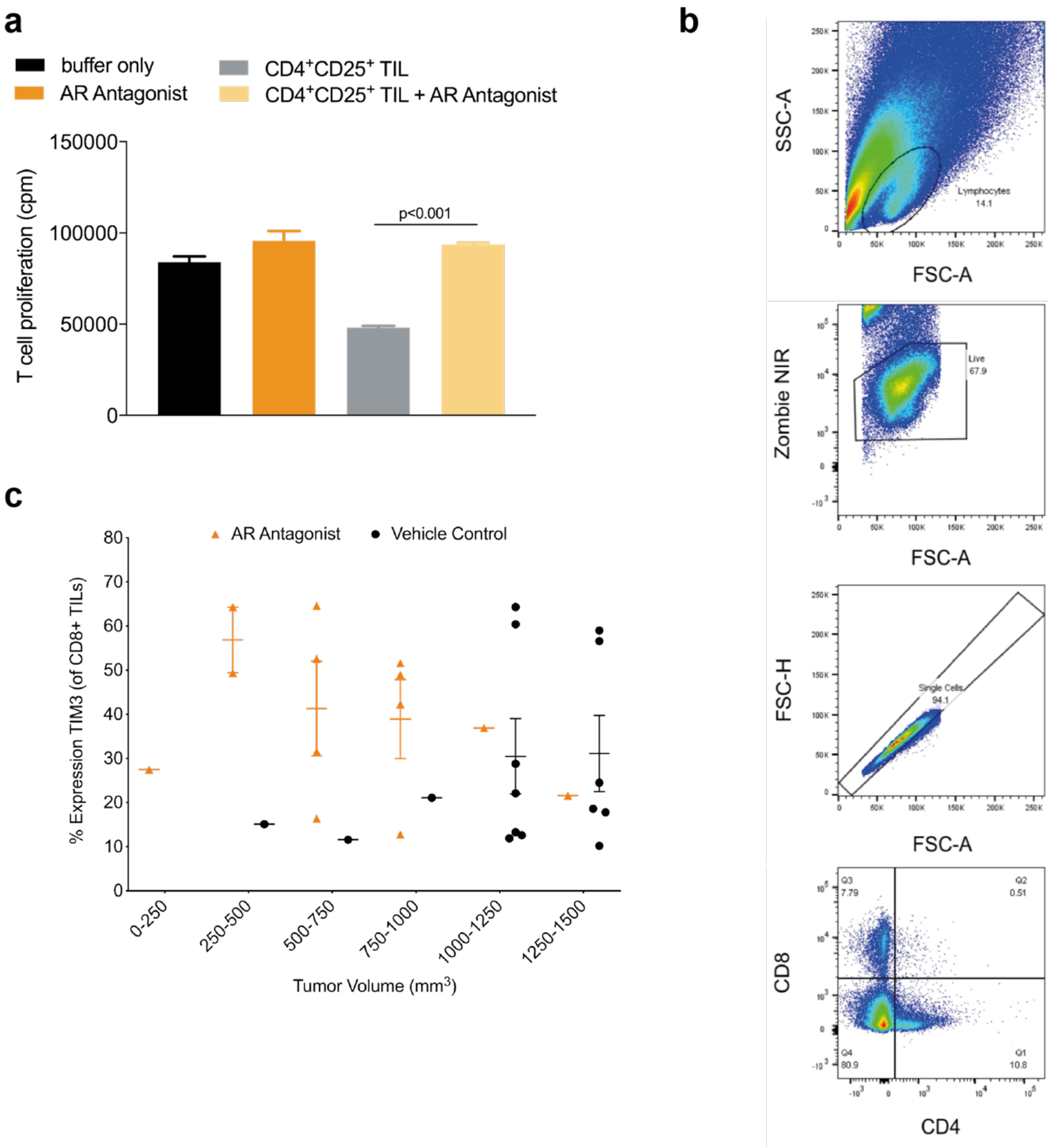

Supplementary figure 1 – supporting data for A2aR antagonist treatment

a $CD4^+CD25^+$ TIL from RencaHA tumor-bearing BALB/c mice were co-cultured with naïve $CD8^+$ CL4 T cells and irradiated mature dendritic cells and K^dHA peptide with or without the A2aR antagonist ZM 241385 at 1.25 µM for 3 days. ^{3}H-thymidine incorporation (cpm) is shown as mean ± SEM. N=10 mice over two experiments (paired t-test). **b** FACS gating strategy for the identification of $CD8^+$ TIL in sequential gates as indicated from the top to the bottom panel. **c** Percentage of $CD8^+$ TIL from RencaHA tumor-bearing BALB/c mice treated with or without the A2aR antagonist ZM 241385 at 10 mg/kg i.p. expressing TIM3 are given as a function of tumor size as mean ± SEM for N=6 experiments.

a

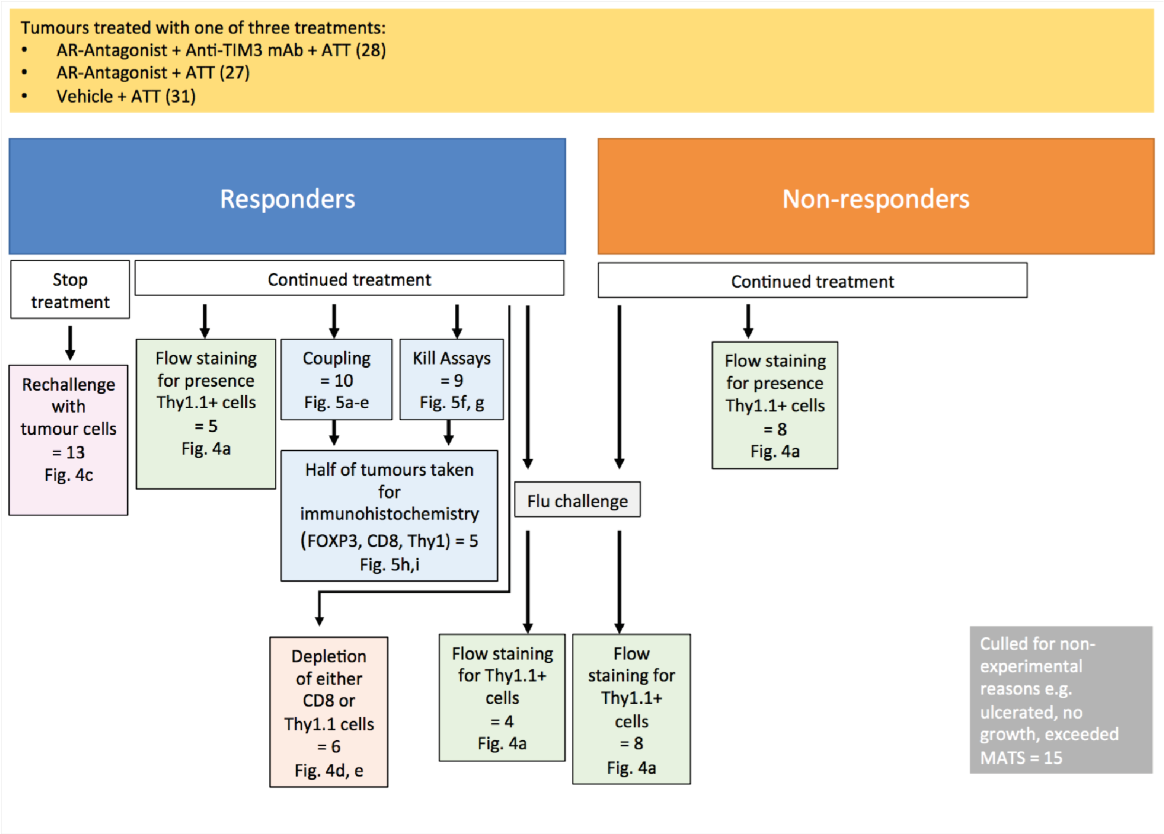

b

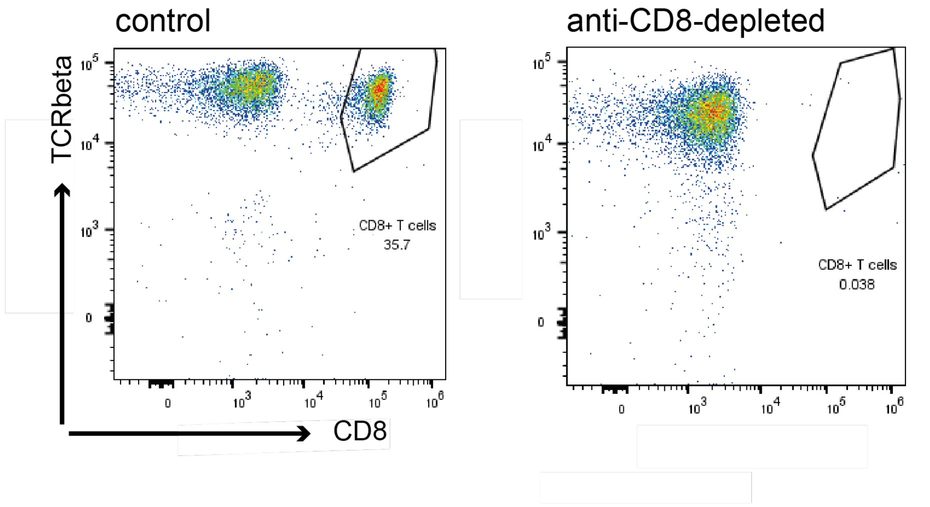

Supplementary figure 2 – Supporting technical data for *in vivo* tumor growth experiments upon adoptive CTL transfer

a Numbers of mice used across the different parts of the experiments in Figs. 4 and 5 are given. **b** The efficiency of the depletion of $CD8^+$ T cells in Fig. 4e is shown in representative FACS plots from blood samples two days after the injection of the depleting antibody or control.
