# Supplementary material for: Adenosine 2A receptor and TIM3 suppress cytolytic killing of tumor cells via cytoskeletal polarization
Source: Commun Biol. 2022 Jan 10;5:9. doi: 10.1038/s42003-021-02972-8 (PMC8748690; doi:10.1038/s42003-021-02972-8)
Supplement: Supplementary file 3 — Description of Additional Supplementary Files [file 42003_2021_2972_MOESM3_ESM.pdf]

## Description of Additional Supplementary Files

**Supplementary Movies:** Representative DIC images of CL4 TILs interacting with Renca target cells in the Page 6 of 17 legends for these must be given in the column to the right. presence of 2μg/ml HA agonist peptide are shown. 20 s intervals in movie acquisition are played back as 2 frames per second.

**File Name:** Supplementary Movie 1
**Description:** Cell coupling occurs in frame 3 (2s indicated movie time). Off interface lamella occur on the top of the interface.

**File Name:** Supplementary Movie 2
**Description:** Cell coupling occurs in frame 7 (4s indicated movie time). Off interface lamella occur on the bottom of the interface.

**File Name:** Supplementary Movie 3
**Description:** Cell coupling occurs in frame 5 (3s indicated movie time). A translocation is shown.

**File Name:** Supplementary Movie 4
**Description:** Cell coupling occurs in frame 5 (2s indicated movie time). A translocation is shown.

**File Name:** Supplementary Data 1
**Description:** Source data for Fig. 2 are given with different tabs for different figure panels.

**File Name:** Supplementary Data 2
**Description:** Source data for Fig. 3 are given with different tabs for different figure panels.

**File Name:** Supplementary Data 3
**Description:** Source data for Fig. 4 are given with different tabs for different figure panels.

**File Name:** Supplementary Data 4
**Description:** Source data for Fig. 5 are given with different tabs for different figure panels.

**File Name:** Supplementary Data 5
**Description:** Source data for Fig. 6 are given with different tabs for different figure panels.

**File Name:** Supplementary Data 6
**Description:** Source data for Fig. 7 except for the calcium data in panel e are given with different tabs for different figure panels.

**File Name:** Supplementary Data 7
**Description:** Source data for Fig. 7e are given.
