# Supplementary material for: Adenosine 2A receptor and TIM3 suppress cytolytic killing of tumor cells via cytoskeletal polarization
Source: Commun Biol. 2022 Jan 10;5:9. doi: 10.1038/s42003-021-02972-8 (PMC8748690; doi:10.1038/s42003-021-02972-8)
Supplement: Supplementary file 15 — Reporting Summary [file 42003_2021_2972_MOESM15_ESM.pdf]

nature research

Corresponding author(s): Christoph Wülfing
David J. Morgan

Last updated by author(s): Oct 20, 2021

Reporting Summary

Nature Research wishes to improve the reproducibility of the work that we publish. This form provides structure for consistency and transparency in reporting. For further information on Nature Research policies, see our Editorial Policies and the Editorial Policy Checklist.

Statistics

For all statistical analyses, confirm that the following items are present in the figure legend, table legend, main text, or Methods section.

n/a	Confirmed	
☐	☒	The exact sample size (*n*) for each experimental group/condition, given as a discrete number and unit of measurement
☐	☒	A statement on whether measurements were taken from distinct samples or whether the same sample was measured repeatedly
☐	☒	The statistical test(s) used AND whether they are one- or two-sided<br>*Only common tests should be described solely by name; describe more complex techniques in the Methods section.*
☐	☒	A description of all covariates tested
☐	☒	A description of any assumptions or corrections, such as tests of normality and adjustment for multiple comparisons
☐	☒	A full description of the statistical parameters including central tendency (e.g. means) or other basic estimates (e.g. regression coefficient) AND variation (e.g. standard deviation) or associated estimates of uncertainty (e.g. confidence intervals)
☒	☐	For null hypothesis testing, the test statistic (e.g. *F*, *t*, *r*) with confidence intervals, effect sizes, degrees of freedom and *P* value noted<br>*Give P values as exact values whenever suitable.*
☒	☐	For Bayesian analysis, information on the choice of priors and Markov chain Monte Carlo settings
☒	☐	For hierarchical and complex designs, identification of the appropriate level for tests and full reporting of outcomes
☒	☐	Estimates of effect sizes (e.g. Cohen's *d*, Pearson's *r*), indicating how they were calculated

Our web collection on statistics for biologists contains articles on many of the points above.

Software and code

Policy information about availability of computer code

Data collection	Image acquisition<br>Volocity, v6.3<br>Leica LASX, v3.7

Data analysis	Flow Cytometry<br>FlowJo, v 10.7.1<br><br>Image analysis<br>Metamorph, v7.7<br>Fiji/ImageJ, v2.1.0<br>Incucyte Zoom, v2018A<br><br>Statistics RStudio v1.0.143 (PCA), SPSS v26, Graphpad Prism v7<br><br>custom additions:<br>A custom-written script to execute the principal component analysis of inhibitory receptor expression is available at GitHub: https://github.com/ge8793/rencaPCA.<br>To obtain measurements of SIL density and spheroid dead volumes, raw data was pre-processed and semi-automatically analysed using a custom-written Cancer Segmentation workflow for the Fiji (52) plugin, MIA (v0.9.26) and its MIA_MATLAB (v1.1.1) package, available at Github via Zenodo: http://doi.org/10.5281/zenodo.2656513 and http://doi.org/10.5281/zenodo.4769615, respectively. The corresponding .mia workflow files are available at https://doi.org/10.5281/zenodo.5511888.

For manuscripts utilizing custom algorithms or software that are central to the research but not yet described in published literature, software must be made available to editors and reviewers. We strongly encourage code deposition in a community repository (e.g. GitHub). See the Nature Research guidelines for submitting code & software for further information.

Data

Policy information about availability of data

All manuscripts must include a data availability statement. This statement should provide the following information, where applicable:

- Accession codes, unique identifiers, or web links for publicly available datasets
- A list of figures that have associated raw data
- A description of any restrictions on data availability

Two large data sets, the flow cytometry data underpinning the principal component analysis to determine inhibitory receptor expression upon A2aR blockade in figure 2 and the spheroid imaging data in figure 6, are accessible through an open data repository of the University of Bristol at https://data.bris.ac.uk/data/ under DOI doi.org/10.5523/bris.11ocsor59owa32ihsxmf0qzj3s. All other source data are provided as supplementary information and corresponding raw imaging data will be made available upon request.

Field-specific reporting

Please select the one below that is the best fit for your research. If you are not sure, read the appropriate sections before making your selection.

☒ Life sciences ☐ Behavioural & social sciences ☐ Ecological, evolutionary & environmental sciences

For a reference copy of the document with all sections, see nature.com/documents/nr-reporting-summary-flat.pdf

Life sciences study design

All studies must disclose on these points even when the disclosure is negative.

Sample size	The Power of in vivo experiments was designed to reach >80%. Experimental group size was determined using the equation: n = (2/(standardized difference2)) x cp,power, where n = sample size per group determined using the formula, d = standardized difference = measurable difference in tumor volume / standard deviation, cp,power = constant for p<0.01 and power at 80% defined using standard Altman's Nomogram = 11.7.
Data exclusions	Mice were censored if tumors were < maximal allowable tumor size (MATS) or if culled for reasons other than tumor size (such as ulceration).<br>Mice bearing tumors > MATS were culled and recorded as dead.<br>Mice were excluded if the tumors didn't grow >3 x 3mm in the first place
Replication	In vivo experiments were carried out over 4 replicates. The direction of effect was the same in all experiments. There was some variation in the magnitude of effect, in line with what is expected in in vivo tumor systems. In vitro experiments were carried out over three experiments and findings were successfully repeated, the range of values is shown in graphs using one point to represent each individual.
Randomization	Mice were allocated randomly into experimental groups by assigning a number to each individual and using a random number generator. Cages contained multiple experimental groups to reduce cage effects.
Blinding	Two out of the four in vivo experimental repeats were carried out double blinded. A person independent to the study made up and labelled the drug reagents as A B and C. One technician assigned mice to groups A, B and C and administered reagents. Another technician measured tumors and was blind to both drug and mouse identity.

Reporting for specific materials, systems and methods

We require information from authors about some types of materials, experimental systems and methods used in many studies. Here, indicate whether each material, system or method listed is relevant to your study. If you are not sure if a list item applies to your research, read the appropriate section before selecting a response.

Materials & experimental systems

n/a	Involved in the study
☐	☒ Antibodies
☐	☒ Eukaryotic cell lines
☒	☐ Palaeontology and archaeology
☐	☒ Animals and other organisms
☒	☐ Human research participants
☒	☐ Clinical data
☒	☐ Dual use research of concern

Methods

n/a	Involved in the study
☒	☐ ChIP-seq
☐	☒ Flow cytometry
☒	☐ MRI-based neuroimaging

Antibodies

Antibodies used

For flow cytometry:
FcBlock no azide (for blockade of Fc receptors) 2.4G2 BD Biosciences 1:50 RRID:AB_2870673
CD8a FITC 53-6.7 BD Bioscience 1:100 RRID:AB_394569
CD8b PeCy7 YTS156.7.7 Biolegend 1:200 RRID:AB_2562777
CD4 AF700 CK1.5 Biolegend 1:100 RRID:AB_493698
CD39 PerCP-Cy5.5 24DMS1 eBioscience 1:100 discontinued
CD73 BV605 TY/11.8 Biolegend 1:100 RRID:AB_2561528
TIM3 PE B8.2C12 Biolegend 1:100 RRID:AB_1626177
TIM3 BV605 RMT3-23 Biolegend 1:100 RRID:AB_2616907
TIGIT APC 1G9 Biolegend 1:100 RRID:AB_10962572
LAG3 PeCy7 C9B7W eBioscience 1:200 discontinued
PD1 BV785 29F.1A12 Biolegend 1:200 RRID:AB_2563680
TCRb AF647 H57-597 Biolegend 1:200 RRID:AB_493346
Thy1.1 FITC OX-7 BD Bioscience 1:100 RRID:AB_395588
Thy1.1 PerCP-Cy5.5 OX-7 Biolegend 1:100 RRID:AB_961438
CEACAM1 APC CC1 Biolegend 1:100 RRID:AB_2632612

For blocking and T cell priming
TIM3 no azide (for in vitro/in vivo blockade) RMT3-23 BioXcell In Vivo mAb in vivo: 100µg/mouse in vitro: 10µg/ml RRID:AB_10949464
Isotype control for anti-TIM3 Rat IgG2a 2A3 no azide (for in vivo/in vitro blockade) BioXcell In Vivo mAb in vivo: 100µg/mouse in vitro: 10µg/ml RRID:AB_1107769
CD8 no azide (for in vivo depletion) 53-5.8 BioXcell InVivoMAb 100µg/mouse RRID:AB_2687706
Thy1.1 no azide (for in vivo depletion) 19E12 BioXcell InVivoMAb 250µg/mouse RRID:AB_2687700
CD3e no azide (for in vitro priming) 145-2C11 BioXcell InVivoMAb 10µg/ml RRID:AB_1107634
CD28 no azide (for in vitro priming) 37.51 BioXcell InVivoMAb 1µg/ml RRID:AB_1107624

For immunohistochemistry:
FcBlock no azide (for blockade of Fc receptors) 2.4G2 BD Biosciences 1:50 RRID:AB_2870673
CD8a no azide 53-6.7 Biolegend 1:500 RRID:AB_312741
Rabbit H+L AF488 Life Technologies 1:1000 RRID:AB_143165
Rabbit H+L AF405 Life Technologies 1:1000 RRID:AB_221605
Rat IgG2a,k Biolegend 1:500 RRID:AB_326523
Rat IgG H+L AF594 ThermoFisher 1:2000 RRID:AB_141374
FOXP3 no azide FJK-16S ThermoFisher 1:100 RRID:AB_467575
FOXP3 APC FJK-16S ThermoFisher 1:40 RRID:AB_469457
Thy1.1 FITC OX-7 BD Bioscience 1:100 RRID:AB_395588
Isotype control for Thy1.1 Mouse IgG1,k FITC BD Bioscience 1:100 RRID:AB_395505
CD19 (dump) BV510 6D5 Biolegend 1:100 RRID:AB_2562136
TCRb SB645 H57-597 ThermoFisher 1:200 RRID:AB_2723704
CD4 PE-Cy5.5 RM4-5 ThermoFisher 1:3000 RRID:AB_1121830
CD8b PE-Cy5 H35-17.2 ThermoFisher 1:3000 RRID:AB_657770
CD25 VioBright-FITC 7D4 Miltenyi 1:200 RRID:AB_2784091
CD73 BV605 TY/11.8 Biolegend 1:100 RRID:AB_2561528
CD39 PerCP-eFluor710 24DMS1 ThermoFisher 1:100 RRID:AB_10717953

Validation

Depletion antibodies were validated as illustrated in supplementary data.
In vivo blocking anti-TIM3 monoclonal antibodies were used in protocols according to published information (sources referenced in the methods in the text).
Validation of the antibodies for use in flow cytometry or immunohistochemistry in mice is available on the manufacturer's (listed

above) websites.

Eukaryotic cell lines

Policy information about cell lines

Cell line source(s)	Murine Renal Carcinoma cell line (RRID:CVCL_2174), ATCC<br>Phoenix retrovirus-producing cell line (RRID:CVCL_H717), Nolan laboratory, Stanford University
Authentication	Murine Renal Carcinoma cell line: staining for MHCI, influenza hemagglutinin neoantigen, ICAM-1, susceptibility to neoantigen-specific killing<br>Phoenix retrovirus-producing cell line: production of MMLV viral particles
Mycoplasma contamination	Tested by PCR
Commonly misidentified lines<br>(See ICLAC register)	N/A

Animals and other organisms

Policy information about studies involving animals; ARRIVE guidelines recommended for reporting animal research

Laboratory animals	Thy1.1+/+ CL4 TCR-transgenic mice [RRID: IMSR_JAX:005307]<br>Thy1.2+/+ BALB/c, (Charles River, Oxford, UK)
Wild animals	N/A
Field-collected samples	N/A
Ethics oversight	All mouse experiments were compliant with UK Home Office Guidelines under PPL 30/3024 to DJM as reviewed by the University of Bristol AWERB (Animal welfare and ethical review body) committee.

Note that full information on the approval of the study protocol must also be provided in the manuscript.

Flow Cytometry

Plots

Confirm that:

- [x] The axis labels state the marker and fluorochrome used (e.g. CD4-FITC).
- [x] The axis scales are clearly visible. Include numbers along axes only for bottom left plot of group (a 'group' is an analysis of identical markers).
- [x] All plots are contour plots with outliers or pseudocolor plots.
- [x] A numerical value for number of cells or percentage (with statistics) is provided.

Methodology

Sample preparation	CTL or TILs, purified as described in the methods section, were resuspended in PBS at a concentration of 1 million cells/1 ml. 500,000 - 1000000 cells for each condition were placed into a polystyrene FACS tube (Corning). Cells were centrifuged and resuspended in 100 µl PBS per tube with 1 µl/100 µl Zombie Aqua or Zombie Near Infared<br>Fixable Live Cell Detection reagent (Biolegend). Tubes were incubated for 15 minutes in the<br>dark at room temperature. Cells were washed in 3 ml FACS buffer and resuspended in 100 µl<br>per tube FcBlock (BDBiosciences) for 15 minutes at 4°C. Cells were washed in 3 ml FACS buffer,<br>pelleted and resuspended in 100 µl FACS buffer per tube with antibody at the required<br>concentration. Cells were incubated for 30 minutes at 4°C. Cells were washed in 3 ml FACS buffer to remove excess antibody before being fixed in 1% paraformaldehyde and analysed.
Instrument	Fortessa X20, BD Influx cell sorter
Software	FlowJo (Treestar), v10.7.1
Cell population abundance	For tumour samples, 12-25% of cells are expected to fall into the lymphocyte gate, of which 50-85% are single, live cells. Sample collection is set so that 10,000 events in the lymphocyte gate (4% of the total 250,000 cell sample) is collected, samples were not considered valid if at least 10,000 CD8+ lymphocytes were not available.

Gating strategy

Boundaries between positive and negative populations were defined using a fluorescence minus one control sample for every stain included in each analysis. This is especially important, as co-inhibitory receptors do not split into positive and negative expression but low, intermediate and high expression on a continuum, therefore populations cannot be accurately defined without FMO controls. The initial FSC/SSC voltages were set to place lymphocytes in the bottom left quadrant of the plots as shown in gating strategy.

☒ Tick this box to confirm that a figure exemplifying the gating strategy is provided in the Supplementary Information.
